# Overexpression of Circular RNA circ_0013587 Reverses Erlotinib Resistance in Pancreatic Cancer Cells Through Regulating the miR-1227/E-Cadherin Pathway

**DOI:** 10.3389/fonc.2021.754146

**Published:** 2021-09-06

**Authors:** Huiting Xu, Runzhi Chen, Qian Shen, Dongmei Yang, Hui Peng, Jin Tong, Qiang Fu

**Affiliations:** ^1^Department of Abdominal Oncology, Hubei Cancer Hospital, Wuhan, China; ^2^Department of Oncology, Tongji Hospital, Tongji Medical College, Huazhong University of Science and Technology, Wuhan, China; ^3^Department of PICC, Tongji Hospital, Tongji Medical College, Huazhong University of Science and Technology, Wuhan, China

**Keywords:** circular RNA, erlotinib, microRNA-1227, E-cadherin, EMT, pancreatic cancer

## Abstract

**Background:**

Erlotinib, a small-molecule epidermal growth factor receptor (EGFR) tyrosine kinase inhibitor, demonstrated therapeutic efficacy against pancreatic cancer. However, acquired resistance to erlotinib in pancreatic cancer is widely observed, and the exact mechanisms have not been fully explored until now. We examined the role of circular RNA circ_0013587 in the acquired resistance to erlotinib in pancreatic cancer cells and explored the underlying mechanisms.

**Methods:**

We selected erlotinib-resistant pancreatic cancer cells from the AsPC-1 cell line. The expression of circ_0013587 was examined by qRT-PCR assays. The effects of circ_0013587 on pancreatic cancer cell proliferation, invasion, and erlotinib resistance were assessed by cell functional assays. Bioinformatic analysis and dual-luciferase reporter assays identified circ_0013587 and E-cadherin as direct targets of miR-1227. Mouse xenograft models were employed to investigate the function of circ_0013587 in erlotinib resistance of tumors *in vivo*.

**Results:**

Circ_0013587 expression was significantly reduced in erlotinib-resistant AsPC-1 cells. We found that increasing circ_0013587 levels in erlotinib-resistant AsPC-1 cells re-sensitized them, whereas reducing circ_0013587 levels in erlotinib-sensitive AsPC-1 cells made them resistant. Mechanically, circ_0013587 released E-cadherin from the suppression of miR-1227, leading to E-cadherin up-regulation. Rescue assays highlighted that circ_0013587 reversed erlotinib resistance in pancreatic cancer cells by increasing E-cadherin levels through reducing the expression of miR-1227. Furthermore, circ_0013587 overexpression sensitized erlotinib-resistant AsPC-1 cells to erlotinib in xenograft models.

**Conclusions:**

Our results demonstrated that down-regulation of circ_0013587 contributes to acquired resistance to erlotinib in pancreatic cancer cells through mediating the miR-1227/E-cadherin pathway and that circ_0013587 is a potential target molecular to overcome erlotinib resistance.

## Background

Pancreatic cancer is a highly aggressive malignancy with a 5-year overall survival rate of only 9%, which is due to its poor response to existing conventional or targeted treatments (1, 2). Epidermal growth factor receptor (EGFR), a transmembrane tyrosine kinase receptor, is frequently dysregulated in various tumors, including pancreatic cancer (3, 4). Up-regulation of EGFR has been linked to poor disease prognosis, invasion, and aggressive clinical behavior of pancreatic cancers (3–5).

Inhibition of EGFR in combination with chemo/radiation therapy has been extensively tested in pancreatic cancer patients (6, 7). Erlotinib is an orally available, reversible tyrosine kinase inhibitor of EGFR (8). Increasing evidence has demonstrated the therapeutic potential of erlotinib as a single agent and in combination with other therapies in the management of pancreatic cancer (6–8). In patients with advanced pancreatic cancer, the addition of erlotinib to gemcitabine improved the overall survival and progression-free survival compared to gemcitabine alone (6, 7). However, in pancreatic cancer patients treated with erlotinib, acquired drug resistance would develop and limit its long-term efficacy (9). Epithelial to mesenchymal transition (EMT) is a pivotal process that plays an important role in maintaining the mesenchymal characteristics of cancer cells. Recent studies have reported that EMT can facilitate the progression and metastasis of pancreatic cancer (10) and drug resistance of cancer cells to Erlotinib (11). There remains an urgent need to further elucidate the mechanism of erlotinib resistance in pancreatic cancer.

MicroRNAs (miRNAs) are small non-coding RNA molecules that act as key mediators in post-transcriptional gene regulation and are involved in the acquired resistance to erlotinib in pancreatic cancer (12, 13). Circular RNAs (circRNAs) are a large class of endogenous non-coding RNA molecules that form a covalently closed-loop (unlike linear RNAs) (14). CircRNAs play crucial roles in a wide range of biological processes related to carcinogenesis, metastasis and chemoresistance (15). CircRNAs exert their biological functions by acting as microRNA (miR) sponges, RNA binding protein sponges, transcriptional regulators and protein templates (14, 15). Circ_0013587 is a circRNA dysregulated in pancreatic cancer tissues in a previous study (16). However, the role and mechanisms of circ_0013587 in the development of erlotinib resistance in pancreatic cancer remain unclear.

Our results showed that circ_0013587 enhances the sensitivity of pancreatic cancer cells to erlotinib by regulating the miR-1227/E-cadherin pathway. Thus, circ_0013587 might be used as a novel potential target to reverse erlotinib resistance for pancreatic cancer patients.

## Materials and Methods

### Collection of Patient-Derived Pancreatic Tissues

This study was reviewed and approved by the Ethics Review Committee at Tongji Hospital, Tongji Medical College, Huazhong University of Science and Technology, China. A set of pancreatic cancer tissues and matched neighboring healthy pancreatic tissue was obtained from pancreatic cancer patients (n = 30) who underwent surgical treatment at Tongji Hospital, Tongji Medical College, Huazhong University of Science and Technology, China. None of these patients have received any preoperative chemotherapy or radiotherapy before surgery. Every patient provided their informed consent before they participated in this study.

### Cell Lines and Cell Culture

Human pancreatic cancer cell lines (BxPc-3, PANC-1, SW1990 and AsPC-1), and the normal pancreatic epithelial cell line HPDE6-C7 were obtained from the American Type Culture Collection (Manassas, VA, USA). Cells were cultured in RPMI-1640 medium (Wako, Osaka, Japan) supplemented with 10% fetal bovine serum (FBS, Gibco, Waltham, MA, USA) and maintained at 37°C with 5% CO2.

### Establishment of Erlotinib-Resistant Cell Line AsPC-1/Erlo

We generated stable erlotinib-resistant cell lines as previously reported (17). In brief, erlotinib-resistant pancreatic cancer cell line AsPC-1/Erlo was developed from the AsPC-1 cell line by exposing the cells to increasing concentrations of erlotinib (Selleck Chemicals, Houston, TX, USA) for 6 months. Cells were cultured in drug-free media for 4-5 days before experiments to prevent acute drug effects.

### Real-Time qPCR Analysis (qRT-PCR)

Total RNA was extracted using TRIzol reagent (Invitrogen, Carlsbad, CA, USA) following the manufacturer’s instructions, and was reverse-transcribed into cDNA as a template for RNA detection using the PrimeScript RT Master Mix (Takara, Dalian, China). For RNase R treatment, total RNA was incubated for 30 min at 37°C with or without 3 U/mg RNase R (Geneseed, China). Preparation of nuclear and cytoplasmic RNA was performed using a Nuclear/Cytoplasmic Isolation kit (BioVision, San Francisco, USA). Quantitative PCR was run with SYBR Premix EX Taq II (Takara Biotechnology, Tokyo, Japan) on an Mx3000P qPCR system (Agilent Technologies, Santa Clara, CA, USA). For circRNA and mRNA, GAPDH was used as an internal control. The expression of miR-1227 was measured using the mirVana qRT-PCR miRNA Detection Kit (Ambion, Austin, TX, USA). In the miRNA RT-PCR reaction, U6 was used as an internal control. The primers used for real-time PCR were purchased from Ribobio (Guangzhou, China).

### Plasmid, siRNA, miRNA Mimics, miRNA Inhibitor and Transfection

The siRNAs, miRNA mimics and miRNA inhibitors used in this study were synthesized by RiboBio (Guangzhou, China). The expression vector encoding E-cadherin was purchased from GeneSeed (Guangzhou, China). For the construction of circ_0013587 overexpression plasmids, the sequence of circ_0013587 was amplified and cloned into a pLCDH-ciR vector by GeneSeed (Guangzhou, China). Stable cells were selected using puromycin (Sigma, St. Louis, MO, USA). Transfection was performed on pancreatic cancer cells using Lipofectamine 3000 reagent (Invitrogen, Carlsbad, CA, USA) according to the manufacturer’s instructions.

### Cell Counting Kit-8 Assay

Cell proliferation and viability was examined using Cell Counting Kit-8 assay (CCK-8, Dojindo, Kumamoto, Japan) as previously reported (18). Pancreatic cancer cells were transfected as indicated and treated with different concentrations of erlotinib for 48 h. Cell survival was determined by the CCK-8 assay.

### Transwell Cell Invasion Assay

Transwell cell invasion assays were conducted as previously described (19). Pancreatic cells were resuspended in serum-free RPMI-1640 medium and then seeded into the upper chamber (8 μm pore size; Millipore, Billerica, MA, USA). A 10% FBS-complete RPMI-1640 medium was added to the lower chamber. After 24 h, the invaded cells to the lower face of the filters were fixed, stained with 0.1% crystal violet solution (Sigma, St. Louis, MO, USA), and counted at ×200 magnification in 10 randomly chosen fields. Experiments were repeated three times.

### Western Blot

Whole-cell protein extractions were prepared with a RIPA buffer (Beyotime, Beijing, China). The proteins were then electro-transferred onto a polyvinylidene difluoride membrane (Millipore, Bedford, MA, USA) and blocked with 5% non-fat milk in Tris-buffered saline. Western blot analysis was performed with commercially available antibodies, anti-E-cadherin (Cell Signaling, MA), anti-Vimentin (Cell Signaling, MA), anti-caspase-3 (Cell Signaling, MA), anti-cleaved caspase-3 (Cell Signaling, MA), anti-Twist (Santa Cruz Biotechnology, Santa Cruz, CA) and anti-β-actin (Santa Cruz Biotechnology, Santa Cruz, CA).

### Dual-Luciferase Reporter Assay

The luciferase reporter vectors containing the full length of E-cadherin 3′-untranslated region (3′-UTR) or circ_0013587 sequences were obtained from RiboBio (Guangzhou, China). The mutant luciferase reporter vectors were generated using a QuikChange site-directed mutagenesis kit (Stratagene, CA, USA). Then, the luciferase reporter plasmids containing wild-type (WT) circ_0013587 fragment, mutant (MUT) circ_0013587 fragment with a mutated miR-1227 binding site, WT E-cadherin 3′-UTR region, or MUT E-cadherin 3′-UTR region with a mutated miR-1227 binding site were co-transfected with miR-1227 mimic, miR-1227 inhibitor or the respective control, along with the Renilla luciferase plasmid pRL-CMV (Promega, WI, USA) using the Lipofectamine 3000 reagent (Invitrogen, Carlsbad, CA, USA). Following 48 h transfection, the Firefly and Renilla luciferase activities were measured using the Dual-Luciferase Reporter Assay System (Promega).

### *In Vivo* Experiment

All procedures were approved by the Animal Research Committee of Tongji Hospital, Tongji Medical College, Huazhong University of Science and Technology, China. The *in vivo* experiments were performed as previously reported (20). In brief, AsPC-1/Erlo cells stably overexpressing circ_0013587 or AsPC-1/Erlo control cells were subcutaneously injected into the right flank of BALB/c nude mice (HFK Bioscience, Beijing, China), respectively. At 1 week post-transplantation, Erlotinib (50 mg/kg) was given every three days through intraperitoneal injection. Tumor volume (V) was monitored by measuring the length (L) and width (W) and calculated with the formula V = (L × W^2^) × 0.5. After 30 days, the mice were sacrificed and the weight of the tumor was recorded.

### Statistical Analysis

Each experiment was performed in triplicate. The results were expressed as the mean ± standard deviation. Student’s t-tests and one-way ANOVA were performed for the comparisons using Prism 6.0 for Windows (GraphPad, San Diego, CA, USA). P < 0.05 was considered statistically significant.

## Results

### Circ_0013587 Expression Is Down-Regulated in Erlotinib-Resistant AsPC-1 Cells

**Figure 1:**
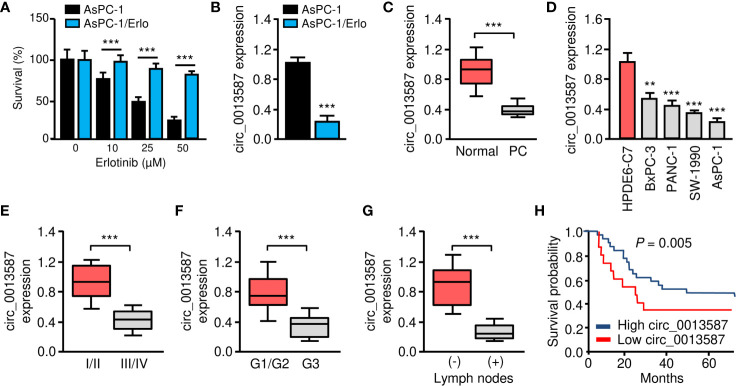
Circ_0013587 expression is down-regulated in erlotinib-resistant AsPC-1 cells. **(A)** Effect of erlotinib treatment (48 h) on the survival of erlotinib-sensitive AsPC-1 cells and erlotinib-resistant AsPC-1/Erlo cells was analyzed using CCK-8 assay. **(B)** The qRT-PCR assay showed significant down-regulation of circ_0013587 expression in AsPC-1/Erlo cells than in AsPc-1 cells. **(C)** qRT-PCR analysis of circ_0013587 levels in pancreatic cancer (PC) and adjacent normal tissues. **(D)** qRT-PCR analysis of circ_0013587 expression in four pancreatic cancer cell lines and a normal pancreatic cell line HPDE6-C7. **(E–G)** The expression of circ_0013587 in pancreatic cancer patients with different tumor stages **(E)**, different tumor grades **(F)**, and patients with (or without) lymph node metastasis **(G)**. **(H)** Kaplan-Meier analysis of overall survival in pancreatic cancer patients with high (above median) *versus* low (below median) circ_0013587 levels. **P < 0.01, ***P < 0.001.

Human pancreatic cancer cell line AsPC-1 harbors KRAS mutation, p53 mutation and wild-type EGFR, thus representing a malignant phenotype commonly observed in pancreatic cancers (17). To understand the mechanisms of acquired erlotinib resistance in pancreatic cancer cells, we selected erlotinib-resistant AsPC-1/Erlo cells by culturing pancreatic cancer cell line AsPC-1 in increasing concentrations of erlotinib. The sensitivity to erlotinib was examined in each cell line using CCK-8 assays. As expected, the AsPC-1/Erlo cells were more resistant than the parental AsPC-1 cells (**Figure 1A**). Our qRT-PCR assay revealed a significant decrease in circ_0013587 expression in AsPC-1/Erlo cells than in AsPC-1 cells (**Figure 1B**). When we compared the expression of circ_0013587 in pancreatic cancer tissues and adjacent normal tissues, we found that the expression of circ_0013587 was significantly lower in pancreatic cancer tissues compared to their counterpart surrounding tissues (**Figure 1C**). Moreover, circ_0013587 levels in pancreatic cancer cell lines were also decreased compared with that in the normal pancreatic epithelial cell line HPDE6-C7 (**Figure 1D**). Notably, circ_0013587 was expressed more lowly in stage III/IV tissues than in stage I/II samples (**Figure 1E**). Those patients with the high-grade disease and lymph node metastasis had significantly lower circ_0013587 expression (**Figures 1F, G**). The prognostic significance of circ_0013587 expression was analyzed in 30 pancreatic cancer patients with the median as the cutoff value. According to the Kaplan-Meier survival curves, the low circ_0013587 group had shorter overall survival than the high circ_0013587 group (**Figure 1H**). Our results demonstrated that reduced circ_0013587 expression may correlate with the acquired erlotinib resistance in pancreatic cancer cells.

### Down-Regulation of circ_0013587 Induces Proliferation, Invasion, and EMT of Pancreatic Cancer Cells *In Vitro*

**Figure 2:**
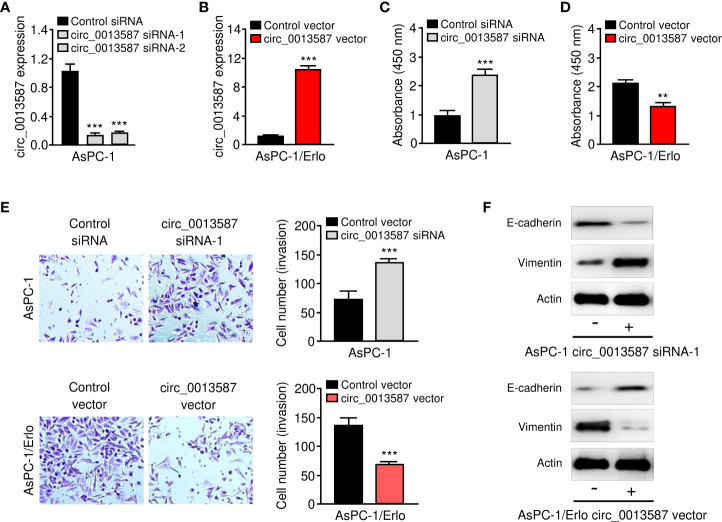
Circ_0013587 represses proliferation and EMT of pancreatic cancer cells *in vitro*. **(A)** Quantification of circ_0013587 levels in AsPC-1 cells transfected with circ_0013587 siRNAs or control siRNA. **(B)** Quantification of circ_0013587 levels in AsPC-1/Erlo cells transfected with circ_0013587 vector or control vector. **(C, D)** CCK-8 assay in pancreatic cancer cells following knockdown **(C)** or overexpression **(D)** of circ_0013587. **(E)** Transwell invasion assays in pancreatic cancer cells after knockdown or overexpression of circ_0013587. **(F)** The expression of E-cadherin and Vimentin was examined in pancreatic cancer cells overexpressing or under-expressing circ_0013587 using western blot analysis. **P < 0.01, ***P < 0.001.

Both loss- and gain-of-function assays were performed to evaluate the effects of circ_0013587 expression on the biological behaviors of pancreatic cancer cells. We knocked down circ_0013587 expression in AsPC-1 cells using siRNAs and also overexpressed circ_0013587 in AsPC-1/Erlo cells using a circ_0013587 overexpression plasmid. Our qRT-PCR results showed that depletion of circ_0013587 with siRNAs resulted in a clear decrease in its levels, and transfection with a circ_0013587 expression plasmid resulted in a robust increase in circ_0013587 levels (**Figures 2A, B**). Cell proliferation and invasion was significantly enhanced following circ_0013587 knockdown, but was significantly attenuated following circ_0013587 overexpression (**Figures 2C–E**). Western blot analysis revealed that knockdown of circ_0013587 in AsPC-1 cells down-regulated E-cadherin protein levels and up-regulated Vimentin protein levels (**Figure 2F**). Conversely, AsPC-1/Erlo cells stably expressing circ_0013587 exhibited higher E-cadherin protein levels and lower Vimentin protein levels compared to control cells (**Figure 2F**). Collectively, these findings suggested that circ_0013587 suppresses proliferation and EMT in pancreatic cancer cells.

### Circ_0013587 Mediates Resistance to Erlotinib in Pancreatic Cancer Cells

**Figure 3:**
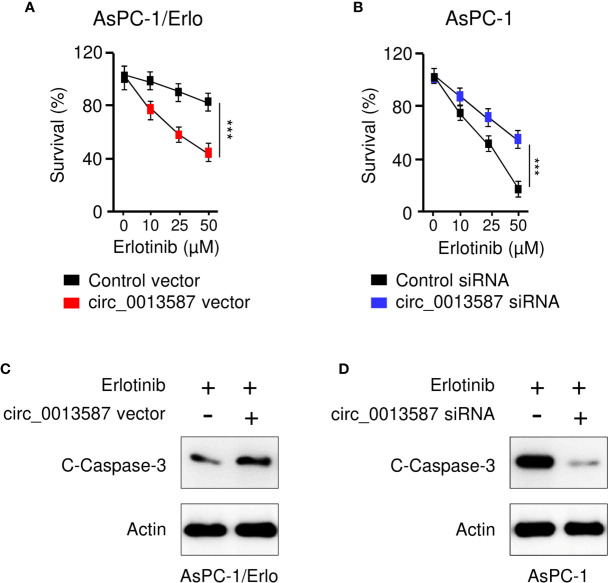
Circ_0013587 mediates resistance to erlotinib in pancreatic cancer cells. **(A)** AsPC-1/Erlo cells transfected with circ_0013587 vector or control vector were exposed to each concentration of erlotinib for 48 h, and the inhibitory effects of erlotinib were evaluated using CCK-8 assays. **(B)** AsPC-1 cells transfected with circ_0013587 siRNA or control siRNA were exposed to each concentration of erlotinib for 48 h, and the inhibitory effects of erlotinib were evaluated using CCK-8 assays. **(C)** Western blot analysis of cleaved **(C)**-caspase-3 in AsPC-1/Erlo cells following overexpression of circ_0013587. **(D)** AsPC-1 cells were transfected with circ_0013587 siRNA or control siRNA and treated with different concentrations of erlotinib for 48 h The expression of cleaved-caspase-3 was determined using western blot analysis. ***P < 0.001.

To evaluate the role of circ_0013587 in acquired resistance to erlotinib, we used the CCK-8 assay to examine the sensitivity of pancreatic cancer to erlotinib after overexpression or knockdown of circ_0013587. The results suggested that circ_0013587 overexpression significantly restored erlotinib sensitivity in AsPC-1/Erlo cells (**Figure 3A**). Following erlotinib treatment, AsPC-1 cells transfected with circ_0013587 siRNA displayed higher viability than those transfected with the siRNA control (**Figure 3B**). Consistently, overexpression of circ_0013587 in AsPC-1/Erlo cells increased the expression level of an apoptosis-related molecule, cleaved caspase-3 (**Figure 3C**). After transfection with circ_0013587 siRNA, cleavage of caspase-3 was inhibited in AsPC-1 cells exposed to erlotinib (**Figure 3D**). These results indicated that up-regulation of circ_0013587 reverses erlotinib resistance in pancreatic cancer cells.

### Circ_0013587 Sponges miR-1227 in Pancreatic Cancer Cells

**Figure 4:**
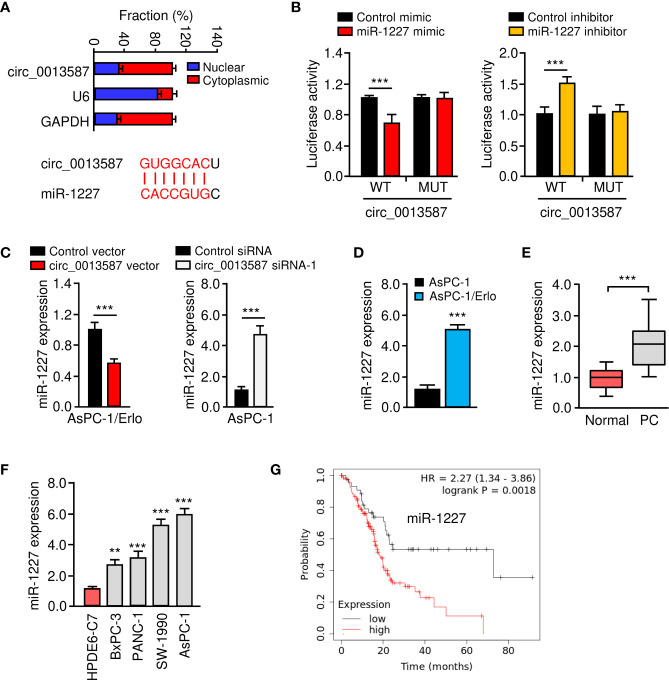
Circ_0013587 sponges miR-1227 in pancreatic cancer cells. **(A)** The qRT-PCR assay showed the cytoplasmic distribution of circ_0013587 in AsPC-1/Erlo cells. Lower panel: the schematic diagram of the predicted miR-1227 binding site for circ_0013587. **(B)** Luciferase activity of WT circ_0013587 or MUT circ_0013587 in AsPC-1/Erlo cells after co-transfection with miR-1227 mimic, and in AsPC-1 cells after co-transfection with miR-1227 inhibitor. **(C)** qRT-PCR analysis of miR-1227 in the cells transfected as indicated. **(D)** qRT-PCR analysis of miR-1227 in AsPC-1/Erlo cells and AsPC-1 cells. **(E)** qRT-PCR analysis of miR-1227 in pancreatic cancer and adjacent normal tissues. **(F)** Measurement of miR-1227 expression in pancreatic cancer cell lines and normal pancreatic cells. **(G)** Kaplan-Meier curves in pancreatic cancer patients with high or low expression of miR-1227. **P < 0.01, ***P < 0.001.

Circ_0013587 was predominantly located in the cytoplasm of AsPC-1/Erlo cells (**Figure 4A**). Using the CircInteractome database (https://circinteractome.nia.nih.gov/), we have bioinformatically predicted miR-1227 as a target of circ_0013587 (**Figure 4A**). Therefore, we further studied the relationship between circ_0013587 and miR-1227 using luciferase reporter assays. As shown in **Figure 4B**, transfection with miR-1227 mimic significantly suppressed the luciferase activity of wild type sequence of circ_0013587, and transfection of miR-1227 inhibitor significantly elevated the luciferase activity of wild type sequence of circ_0013587 (**Figure 4B**). Meanwhile, these effects of miR-1227 mimic or inhibitor were significantly abolished in mutant circ_0013587 plasmids with the mutant sequence in the binding site of miR-1227 (**Figure 4B**). These results supported a direct interaction between miR-1227 and circ_0013587. Furthermore, qRT-PCR results demonstrated that overexpression of circ_0013587 decreased, while knockdown of circ_0013587 increased miR-1227 levels in pancreatic cancer cells (**Figure 4C**). Using qRT-PCR assays, we found that the expression of miR-1227 was significantly higher in AsPC-1/Erlo cells than AsPC-1 cells (**Figure 4D**). We confirmed the up-regulation of miR-1227 in pancreatic cancer samples compared to normal tissues (**Figure 4E**). The levels of miR-1227 in pancreatic cancer cell lines were clearly higher than that in normal cells (**Figure 4F**). Using the Kaplan-Meier Plotter database (http://kmplot.com/analysis/), our survival analysis suggested that higher miR-1227 expression was correlated with a worse outcome in pancreatic cancer patients (**Figure 4G**). These findings suggest that circ_0013587 directly sponges miR-1227 in pancreatic cancer cells.

### Circ_0013587 Mediates Resistance to Erlotinib Through Regulating miR-1227 Expression

**Figure 5:**
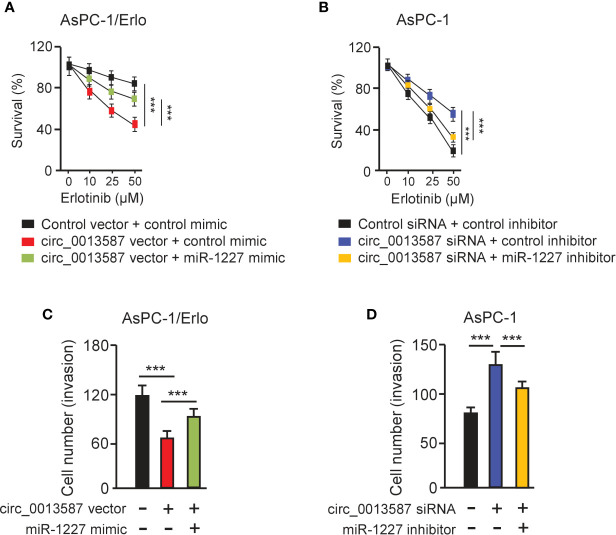
Circ_0013587 mediates erlotinib resistance through regulating miR-1227 expression. **(A, B)** AsPC-1/Erlo **(A)** and AsPC-1 **(B)** cells transfected as indicated were exposed to each concentration of erlotinib for 48 h, and the inhibitory effects of erlotinib were evaluated using CCK-8 assays. **(C, D)** Pancreatic cancer cells were transfected as indicated and cell invasion was measured using transwell invasion assays. ***P < 0.001.

CCK-8 assays showed that overexpression of circ_0013587 sensitized resistant AsPC-1/Erlo cells to erlotinib, while this effect was reversed by restoration of miR-1227 expression (**Figure 5A**). In line with this result, suppression of circ_0013587 contributed to erlotinib resistance in AsPC-1 cells, and transfection with miR-1227 inhibitor was able to overcome erlotinib resistance induced by circ_0013587 silencing (**Figure 5B**). Then, we examined whether the effects of circ_0013587 on cell invasion are mediated by miR-1227 in pancreatic cancer cells. Cell invasion assays revealed that overexpression of circ_0013587 suppressed cell invasion, but cell invasion ability was partially rescued by miR-1227 overexpression (**Figure 5C**). Down-regulation of circ_0013587 increased cell invasion, and this promotion was reversed by a miR-1227 inhibitor (**Figure 5D**). These results demonstrated that up-regulation of circ_0013587 reverses acquired resistance to erlotinib and suppresses cell invasion in pancreatic cancer cells through mediating miR-1227 expression.

### E-Cadherin Is a Direct Target Gene of miR-1227

**Figure 6:**
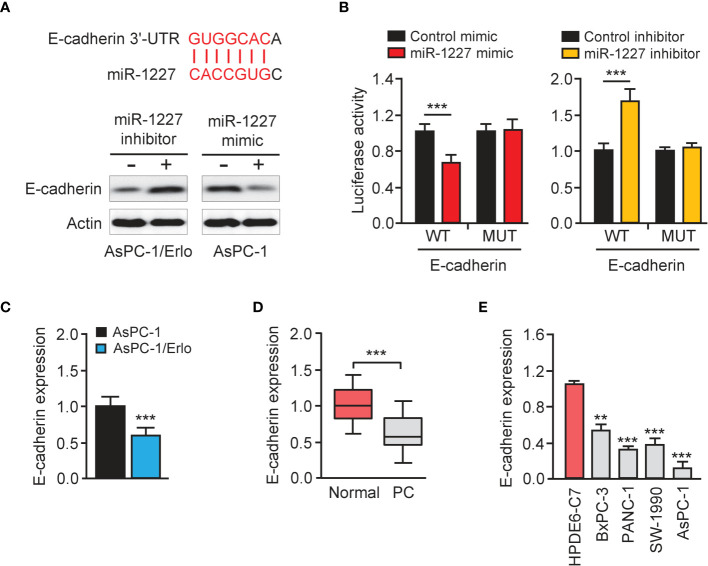
E-cadherin is a direct target gene of miR-1227. **(A)** Upper panel: schematic diagram of the predicted miR-1227 binding site for *E-cadherin* 3′-UTR. Bottom panel: western blot analysis of E-cadherin expression in pancreatic cancer cells transfected as indicated. **(B)** Luciferase activity of WT or MUT *E-cadherin* 3′-UTR in AsPC-1 cells after co-transfection with miR-1227 mimic, and in AsPC-1/Erlo cells after co-transfection with miR-1227 inhibitor. **(C)** qRT-PCR analysis of E-cadherin expression in AsPC-1/Erlo and AsPC-1 cells. **(D)** qRT-PCR analysis of E-cadherin expression in pancreatic cancer and normal tissues. **(E)** qRT-PCR analysis of E-cadherin expression in pancreatic cancer cell lines and normal cells. **P < 0.01, ***P < 0.001.

We queried the TargetScan database (http://www.targetscan.org/vert_72/) to identify miR-1227 target genes and discovered a highly-conserved miR-1227 binding site on the 3′-UTR of E-cadherin (**Figure 6A**, upper). The western blot assay revealed a reduction in E-cadherin protein expression in AsPC-1 cells harboring miR-1227 overexpression (**Figure 6A**, bottom). Transfection with miR-1227 inhibitor increased E-cadherin levels in AsPC-1/Erlo cells (**Figure 6A**, bottom). The relative luciferase activity of wild-type E-cadherin 3′-UTR was significantly down-regulated in AsPC-1 cells transfected with miR-1227 mimic than in cells transfected with control mimic (**Figure 6B**). There was a significantly higher relative luciferase activity of wild-type E-cadherin 3′-UTR in AsPC-1/Erlo cells transfected with miR-1227 inhibitor than in cells transfected with control inhibitor (**Figure 6B**). However, the introduction of miR-1227 mimic or inhibitor did not significantly impact the relative luciferase activity of mutant E-cadherin 3′-UTR (**Figure 6B**). We also verified the down-regulation of E-cadherin in AsPC-1/Erlo cells than AsPC-1 cells (**Figure 6C**). The levels of E-cadherin were significantly lower in pancreatic cancer tissues compared with normal tissues (**Figure 6D**). Pancreatic cancer cells expressed lower expression of E-cadherin than normal cells (**Figure 6E**). The findings suggested that miR-1227 can bind to and suppress the expression of E-cadherin.

### MiR-1227 Enables Erlotinib Resistance and Cell Invasion *via* Reducing E-Cadherin Expression

**Figure 7:**
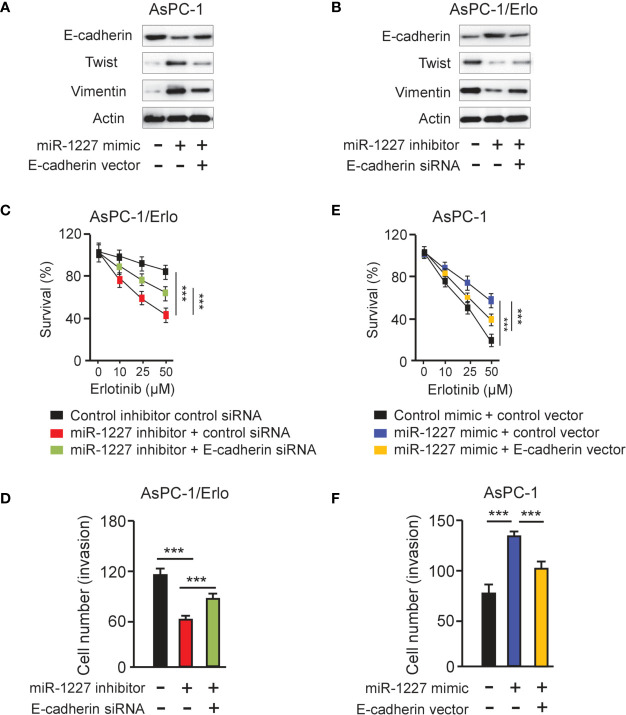
MiR-1227 promotes erlotinib resistance and cell invasion *via* reducing E-cadherin expression. **(A, B)** Western blot analysis of E-cadherin, Twist, and Vimentin in AsPC-1 **(A)** and AsPC-1/Erlo **(B)** cells transfected as indicated. **(C)** AsPC-1/Erlo cells transfected as indicated were exposed to each concentration of erlotinib for 48 h, and the inhibitory effects of erlotinib were evaluated using CCK-8 assays. **(D)** AsPC-1/Erlo cells were transfected as indicated, and cell invasion was evaluated using cell invasion assays. **(E)** AsPC-1 cells transfected as indicated were exposed to each concentration of erlotinib for 48 h, and the inhibitory effects of erlotinib were evaluated using CCK-8 assays. **(F)** AsPC-1 cells were transfected as indicated, and cell invasion was evaluated using cell invasion assays. ***P < 0.001.

To access whether miR-1227 regulates erlotinib resistance and cell invasion by modulating E-cadherin expression, we examined the protein levels of E-cadherin, Twist, and Vimentin in AsPC-1 cells co-transfected with miR-1227 mimic (or control mimic) along with E-cadherin expression vector (or control vector), and in AsPC-1/Erlo cells co-transfected with miR-1227 inhibitor (or control inhibitor) along with E-cadherin siRNA (or control siRNA). Our results suggested that miR-1227 mimic induced the expression of Twist and Vimentin, and decreased E-cadherin expression (**Figure 7A**). These impacts of miR-1227 overexpression were largely abolished by ectopic overexpression of E-cadherin in AsPC-1 cells (**Figure 7A**). In addition, inhibition of miR-1227 markedly increased E-cadherin levels and reduced the protein expression of Twist and Vimentin (**Figure 7B**). Co-transfection with E-cadherin siRNA reversed these changes in AsPC-1/Erlo cells (**Figure 7B**). Our CCK-8 and cell invasion assays demonstrated that miR-1227 inhibitor-induced suppression of erlotinib resistance and cell invasion was promoted by E-cadherin knockdown (**Figures 7C, D**). Also, miR-1227 overexpression facilitated erlotinib resistance and cell invasion in AsPC-1 cells, whereas forced overexpression of E-cadherin could significantly suppress erlotinib resistance and cell invasion induced by miR-1227 mimic (**Figures 7E, F**). These findings demonstrated that miR-1227 mediates the acquired resistance to erlotinib and invasive ability of pancreatic cancer cells *via* E-cadherin.

### Overexpressing circ_0013587 Expression Enhanced Erlotinib Sensitivity in Erlotinib-Resistant AsPC-1/Erlo Cell Mouse Xenografts

**Figure 8:**
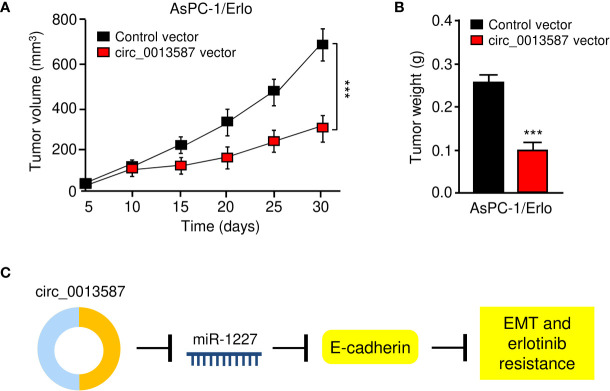
Overexpression of circ_0013587 reverses erlotinib resistance in pancreatic cancer *in vivo*. **(A)** Mice were injected with AsPC-1/Erlo cells stably expressing circ_0013587 or AsPC-1/Erlo control cells and were treated with erlotinib. Tumor growth was measured every 5 days to draw the growth curve. **(B)** Tumor weight was recorded for each derived xenograft tumor. **(C)** A schematic model showing that circ_0013587 reverses erlotinib resistance in pancreatic cancer cells by regulating the miR-1227/E-cadherin pathway. ***P < 0.001.

Nude mouse xenograft models were established using AsPC-1/Erlo cells stably expressing circ_0013587 or AsPC-1/Erlo control cells. Both groups of nude mice received an intraperitoneal injection of erlotinib. As expected, erlotinib alone failed to suppress tumor growth *in vivo* (**Figures 8A, B**). However, the tumor volume and weight of the circ_0013587-expressing AsPC-1/Erlo cell group was significantly lower than the control group (**Figures 8A, B**). These results suggested that overexpression of circ_0013587 reversed erlotinib resistance in pancreatic cancer *in vivo*. Collectively, these results indicated that circ_0013587 sponges miR-1227 to enhance E-cadherin level, in turn attenuating the acquired resistance to erlotinib and invasive ability of pancreatic cancer cells (**Figure 8C**).

## Discussion

Previous studies have established that the employment of EGFR tyrosine-kinase inhibitors (EGFR-TKIs) (such as erlotinib) have achieved encouraging progress in treating patients with pancreatic cancer, unfortunately, after the initial response, many patients would inevitably acquire resistance to erlotinib (9). Several resistance mechanisms to EGFR-TKIs have been demonstrated, including a secondary point mutation in codon 790 of exon 20 (T790M) of the EGFR gene, EGFR gene amplification, MET gene amplification, HER2 gene amplification and EMT (21, 22). Mesenchymal status is closely related to the resistance to erlotinib in pancreatic cancer (23) and lung cancer (24, 25). A previous study using siRNA to silence ZEB-1, a key EMT inducer, has shown that ZEB-1 silencing enhanced sensitivity to erlotinib in pancreatic cancer cells (10). Twist is another transcription factor responsible for EMT in cancer (26). In pancreatic cancer, deletion of Twist led to enhanced sensitivity to erlotinib treatment (27). In addition, although aberrant expression of circRNAs has been implicated in multiple aspects of cancer pathophysiology such as proliferation, EMT and invasion (28), their specific roles and detailed mechanisms in regulating erlotinib resistance in pancreatic cancer remain incompletely revealed.

In this study, we delineated the critical role of circ_0013587 in regulating EMT and the sensitivity of pancreatic cancer cells to erlotinib. Our data provided several advanced insights into the functions of circ_0013587 and its underlying mechanisms: (i) circ_0013587 expression is down-regulated in erlotinib-resistant AsPC-1/Erlo cells; (ii) circ_0013587 suppresses the EMT process and sensitizes pancreatic cancer cells to erlotinib treatment; (iii) circ_0013587 acts as a competing endogenous RNA for miR-1227 to up-regulate E-cadherin expression; (iv) up-regulation of miR-1227 and down-regulation of E-cadherin contributes to increased resistance to erlotinib in pancreatic cancer cells; and (v) circ_0013587 reverses erlotinib resistance in pancreatic cancer *in vivo*. Therefore, our results highlight an underlying role of circ_0013587 repression in pancreatic cancer drug response and identify circ_0013587 as an attractive target to overcome erlotinib resistance.

CircRNAs have been shown to regulate several of the hallmarks of cancer (29). For instance, circHIPK3 exerts critical oncogenic roles in promoting the EMT features of pancreatic cancer cells (30). Another study suggested that circNEIL3 regulates the expression of ADAR1 by sponging miR-432-5p to induce RNA editing of glioma-associated oncogene 1, ultimately promoting EMT in pancreatic cancer (31). What interested us was that circ_0013587 has been reported as a circRNA down-regulated in pancreatic cancer tissues compared with normal tissues (16). However, circ_0013587 has never been linked to EMT and erlotinib resistance in pancreatic cancer. Our data first confirmed that the expression of circ_0013587 was significantly down-regulated in pancreatic cancer samples, and its down-regulation was associated with poorer patient prognosis. Intriguingly, we verified that re-expression of circ_0013587 could not only inhibit cell proliferation, but also suppress cell invasion and the EMT process. Then, we found that overexpression of circ_0013587 overcome erlotinib resistance *in vitro* and *in vivo*. Collectively, our data demonstrated that circ_0013587 exhibited tumor suppressor properties in pancreatic cancer cells. Also, we confirmed that the use of circ_0013587-targeted clinical approach may be able to attenuate pancreatic cancer progression and overcome erlotinib resistance.

Existing literature pointed out that circRNAs could exert their functions in cancers by acting as a competing endogenous RNA for miRNAs (14, 15). CircRNAs could bind with miRNAs, thereby freeing mRNA targets from the regulation of miRNAs (14, 15). This study revealed that although circ_0013587 was mainly localized in the cytoplasm of erlotinib-resistant AsPC-1/Erlo cells, indicating that circ_0013587 may work as a competing endogenous RNA. Then, our experiments uncovered a direct interaction between circ_0013587 and miR-1227 in pancreatic cancer cells. Our study extended the regulatory mechanism for circ_0013587 function, by which circ_0013587 represses the invasive ability and erlotinib resistance in pancreatic cancer cells, at least in part through competitively binding miR-1227. However, the underlying mechanisms of circ_0013587 remain to be fully explored in our future research.

A few studies of miR-1227 in human malignancies have been published (32–34). For example, long non-coding RNA OR3A4 promotes osteosarcoma cell proliferation and invasion by sponging miR-1227 (32). A recent report indicated that circRNA circTNFRSF21 was highly expressed in endometrial cancer tissues, and circTNFRSF21 was able to accelerate endometrial cancer cell growth, proliferation and *in vivo* tumor formation (33). Mechanically, circTNFRSF21 acts as a sponge of miR-1227 to rescue MAPK13/ATF2 signaling pathway activity in endometrial cancer cells (33). These results implied the tumor-suppressor roles of miR-1227 in these tumors. However, a former study supported that miR-1227 increased cell proliferation and invasion in osteosarcoma (34). Here, we provided interesting data that miR-1227 served as a promoter of invasion and erlotinib resistance in pancreatic cancer cells. This inconsistency may suggest that the roles of miR-1227 in human tumors might depend on cell type and intracellular context.

E-cadherin is a type of calcium-dependent transmembrane glycoprotein that mediates cell-cell adhesion between epithelial cells (35). A recent study reported that E-cadherin expression was completely intact and strongly positive in all normal pancreatic tissues, but was mostly weak and focally lost in pancreatic cancer tissues (35). Knockdown of E-cadherin enhanced the migration and invasion capacity of pancreatic cancer cells (36). Another study demonstrated a causal role for E-cadherin in maintaining an epithelial phenotype of pancreatic cancer cells (37). Importantly, ectopic expression of E-cadherin was shown to overcome resistance to erlotinib in pancreatic cancer (38). Similarly, restoration of E-cadherin expression significantly increases the sensitivity to epidermal growth factor receptor inhibitor gefitinib in lung cancer cells (39). In human breast epithelial cells, shRNA-mediated loss of E-cadherin resulted in up-regulation of expression of Twist and Vimentin (40). However, it is still unknown if E-cadherin has any relevance in mediating Twist and Vimentin expression in pancreatic cancer. In the present study, we found that overexpression of E-cadherin could enhance the sensitivity of pancreatic cancer cells to erlotinib possibly by regulating Twist and Vimentin expression. Of note, Twist is a transcriptional repressor of E-cadherin in breast cancer (41). Further studies are required to investigate whether Twist can form a feedback loop with E-cadherin, resulting in the gain of cell invasiveness and increased erlotinib resistance of pancreatic cancer cells.

However, there were limitations in this study. First, the samples number was relatively small. Second, although possible mechanisms for circ_0013587 have been explored in AsPC-1/Erlo and AsPC-1 cells, and further validation would be performed in other representative pancreatic cancer cell lines.

## Conclusion

In conclusion, our work shows that down-regulation of circ_0013587 confers chemoresistance of pancreatic cancer cells to erlotinib *via* mediating the miR-1227/E-cadherin pathway. Our findings provide insight into the circ_0013587/miR-1227/E-cadherin axis as a promising therapeutic target against erlotinib-resistant pancreatic cancer, implying important translational implications.

## Data Availability Statement 

The original contributions presented in the study are included in the article/supplementary material. Further inquiries can be directed to the corresponding author.

## Ethics Statement 

The studies involving human participants were reviewed and approved by the Ethics Review Committee at Tongji Hospital, Tongji Medical College, Huazhong University of Science and Technology, China. The patients/participants provided their written informed consent to participate in this study. The animal study was reviewed and approved by the Animal Research Committee of Tongji Hospital, Tongji Medical College, Huazhong University of Science and Technology, China.

## Author Contributions 

QF supervised the study. HX conducted the experiments. RC, QS, DY, HP, and JT analyzed the results. HX and QF wrote the manuscript. All authors contributed to the article and approved the submitted version.

## Funding

This study was supported by the National Natural Science Foundation of China (No. 81974381).

## Conflict of Interest

The authors declare that the research was conducted in the absence of any commercial or financial relationships that could be construed as a potential conflict of interest.

## Publisher’s Note

All claims expressed in this article are solely those of the authors and do not necessarily represent those of their affiliated organizations, or those of the publisher, the editors and the reviewers. Any product that may be evaluated in this article, or claim that may be made by its manufacturer, is not guaranteed or endorsed by the publisher.
